# A Case of Triplets

**Published:** 1876-02

**Authors:** 


A Case of Triplets.—Dr. J. B. Robertson (*Pacific Medical and Surgical Journal*) reports such a case. He says: "The doctor being called at 9 P.M., October 15th, found Mrs. L. apparently in labor, but with a loss of power of uterine contraction amounting very nearly to inertia of the uterus. He gave ergot in fifteen-grain doses every half hour until the womb regained its power of contraction. The triplets were born the next day. They were all females; weight as follows: $5\frac{1}{2}$ lbs., $6\frac{1}{2}$ lbs., 6 lbs.: total, 18 lbs. We estimated the total weight which the patient carried to be about thirty pounds, including the anniotic fluid, etc. A point worthy of notice is the presentations, all being of the head, the vertex of each child presenting. Another noteworthy point has relation to the placentæ, which I had the opportunity of examining. There were two placentas, the larger having one umbilical cord, which the doctor says was attached to the first child. The smaller had two cords attached. The two placentæ were connected by narrow, irregularly shaped placental lobes, together with lamellated albuminous tissue. The placental blood-vessels could also be distinctly observed running from one placenta to the other. There were three seperate membranes or bags of water, each of which required to be ruptured. Mrs. L. felt considerable exhausted after labor, but did well, and was soon able to attend to household duties. She had only the usual amount of flooding, which, I believe, is exceptional, as it is stated by most obstetrical writers that 'hemorrhage is always to be suspected in plural births.' The after-pains lasted twenty hours and were quite severe at first. Nov. 24th, the three little girls are living, and seem to be healthy children."

